# Proteomic antibacterial characterization of flavonoid xanthohumol and probiotic *Clostridium butyricum* on pathogenic *Clostridioides difficile*

**DOI:** 10.1186/s13020-026-01343-x

**Published:** 2026-02-03

**Authors:** Shenkun Wei, Guorong Li, Qiong Yang, Xinping Zhu, Junjie Liang, Mengyi Liu, Zijian Chen, Xia Liu, Jun-Yu Xu, Wei Chen

**Affiliations:** 1 School of Chinese Materia Medica, Nanjing University of Chinese Medicine, Nanjing, Jiangsu China; 2 Zhongshan Institute for Drug Discovery, Shanghai Institute of Materia Medica, Chinese Academy of Sciences, Zhongshan, Guangdong China; 3 Department of Urology, The First Affiliated Hospital of Wenzhou Medical University, Wenzhou, China; 4 State Key Laboratory of Drug Research, Shanghai Institute of Materia Medica, Chinese Academy of Sciences, Shanghai, China

**Keywords:** *Clostridioidesdifficile*, *Clostridiumbutyricum*, Glycolysis, Post-translational modification, Xanthohumol

## Abstract

**Background:**

The management of dysbiotic gut microbiota in *Clostridioides difficile* infection has attracted increasing scholarly attention. The development of therapeutic agents with low toxicity, derived from both the flavonoid xanthohumol and the short-chain fatty acid-producing probiotic *Clostridium butyricum*, holds considerable promise for combating *Clostridioides difficile* infection. Despite their therapeutic potential, the molecular mechanisms underlying the anti-*Clostridioides difficile* effects remain inadequately characterized.

**Methods:**

In this study, we established a dextran sulfate sodium-induced inflammatory model using Caco-2 intestinal epithelial cells. The protective effects of xanthohumol against *Clostridioides difficile* infection superimposed on colitis were evaluated through cell viability assays, analysis of inflammatory signaling pathways, and proteomic profiling. Subsequent in vitro assays and proteomic analyses were conducted to assess the influence of xanthohumol and *Clostridium butyricum* supernatant on *Clostridioides difficile*. Furthermore, tandem mass tag-based post-translational modification proteomics was employed to elucidate the underlying molecular mechanisms and key pathways. Finally, critical metabolic enzyme activity assays were performed to validate the regulatory roles of these pathways.

**Results:**

Xanthohumol significantly alleviated *C. difficile*-induced damage in Caco-2 cells, enhanced cell viability, and suppressed the activation of inflammatory signaling pathways. In vitro experiments demonstrated that both xanthohumol and *C. butyricum* supernatant reduced bacterial colonization, inhibited growth, and attenuated toxin production. Proteomic analyses revealed substantial alterations in the proteome of *C. difficile* in response to each treatment. Post-translational modification proteomics further indicated that both treatments modulate lysine acetylation levels, influencing glycolysis pathways and ultimately diminishing the pathogen’s virulence. Furthermore, mass spectrometry identified a specific lysine acetylation at the K280 site of fructose-1,6-bisphosphate aldolase, a key enzyme in glycolysis. Functional validation via site-directed mutagenesis confirmed the essential role of this acetylation in regulating the catalytic activity of fructose-1,6-bisphosphate aldolase.

**Conclusions:**

Our study demonstrates that xanthohumol and *Clostridium butyricum* attenuate the pathogenicity of *Clostridioides difficile* through modulation of lysine acetylation and disruption of glycolysis metabolism. These findings highlight their potential as promising therapeutic strategies for treating *Clostridioides difficile* infection.

**Graphical Abstract:**

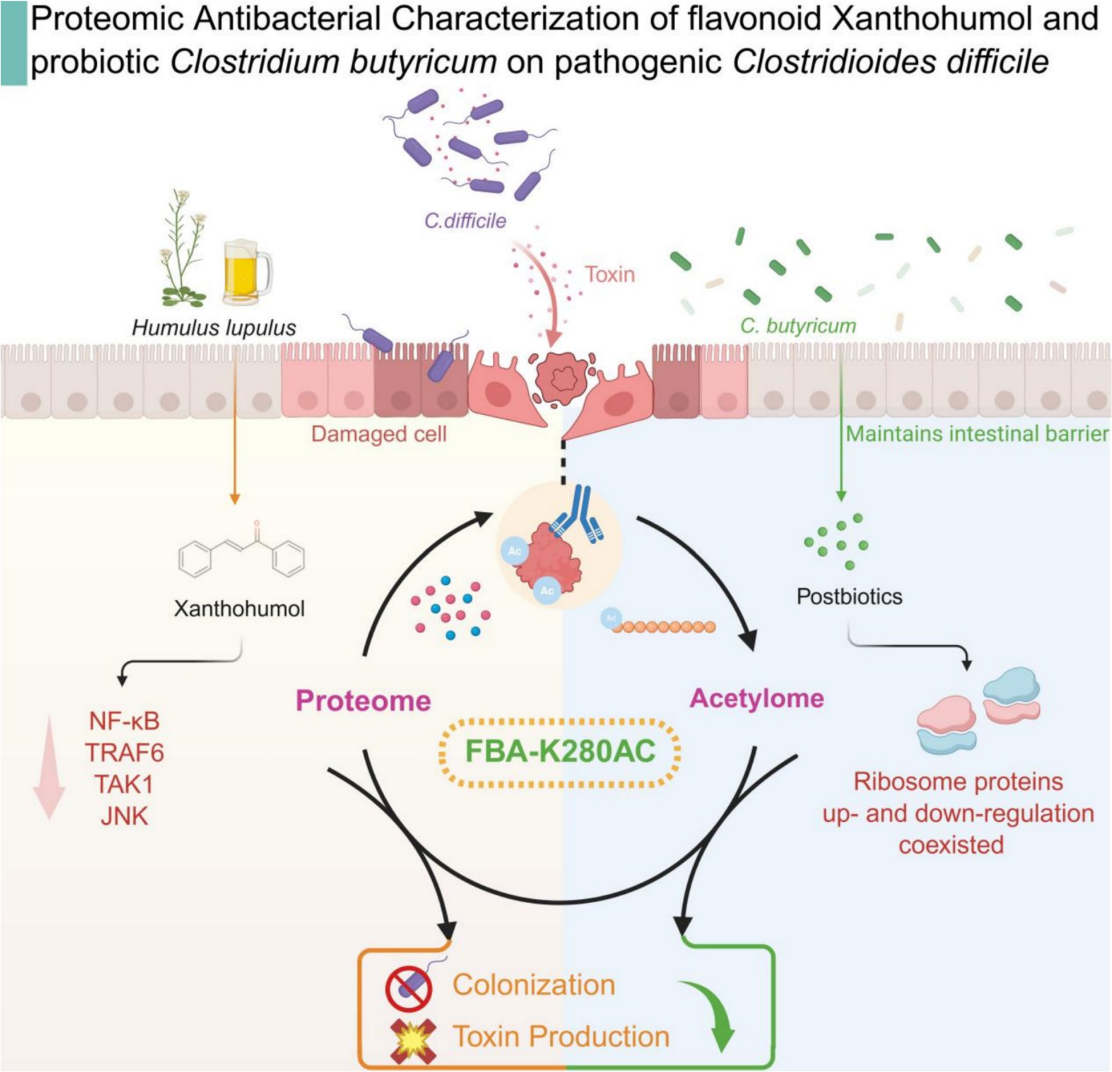

**Supplementary Information:**

The online version contains supplementary material available at 10.1186/s13020-026-01343-x.

## Background

*Clostridioides difficile* (*C. difficile*), a Gram-positive bacterium, has emerged as a leading nosocomial pathogen of global importance [1, 2]. Epidemiological evidence indicates a significant rise in the incidence of *C. difficile* infection (CDI) over the past two decades, which accounts for 15–20% of cases of antibiotic-associated diarrhea and presents with a clinical spectrum ranging from mild diarrhea to severe pseudomembranous colitis [3]. This trend imposes considerable burdens on healthcare systems worldwide. Patients with inflammatory bowel disease (IBD), characterized by chronic intestinal dysbiosis and immune dysfunction, exhibit a particularly increased susceptibility to CDI [4]. A well-documented bidirectional pathological relationship exists between CDI and IBD. CDI not only constitutes a prevalent infectious complication among IBD patients but also acts as a catalyst for disease exacerbation [5]. Clinical outcomes are notably poorer in IBD patients with concurrent CDI, characterized by heightened risks of colectomy and increased mortality rates [6]. Consequently, targeted therapeutic strategies against *C. difficile* may provide significant advantages in the management of IBD. However, current first-line treatments, such as vancomycin, metronidazole, and fidaxomicin, encounter increasing challenges due to emerging resistance patterns and the continuous evolution of the pathogen [7].

Bioactive compounds sourced from medicinal plants and microorganisms present a promising alternative therapeutic strategy for combating CDI. In particular, phytochemical constituents isolated from various medicinal plants have shown considerable potential for CDI intervention, with flavonoids exhibiting notably potent anti-*C. difficile* activity. Xanthohumol (XN), a bioactive prenylated flavonoid derived from *Humulus lupulus* [8], has attracted substantial scientific interest due to its broad-spectrum pharmacological properties, including antimicrobial [9], anticancer [10], and anti-inflammatory activities [11]. Emerging evidence suggests that XN is a promising therapeutic candidate against *C. difficile*, as it demonstrates selective inhibition of the pathogen while preserving gut microbiota balance [12]. In vivo studies using rat models of colitis have confirmed the impact of XN administration on CDI progression [13], with reported minimum inhibitory concentration and minimum bactericidal concentration values against toxigenic *C. difficile* strains exhibiting potency comparable to that of conventional antibiotics [9].

The gut microbiota orchestrates a complex, multi-tiered antimicrobial defense system through its metabolic products. Short-chain fatty acids (SCFAs) serve as fundamental agents providing broad-spectrum antimicrobial protection, whereas secondary bile acids offer more selective, targeted inhibition. Furthermore, amino acid metabolites facilitate highly specific molecular regulation. This hierarchical antimicrobial framework, which transitions from basic defense to precise modulation, provides significant insights for the development of novel anti-infective therapies [14, 15]. SCFAs, as microbial metabolites produced by the gut microbiota, are crucial in modulating host immune homeostasis and maintaining intestinal barrier integrity [16, 17]. Butyrate, a primary fermentation end product analogous to lactate, is considered one of the most physiologically significant SCFAs [18, 19]. Upon rapid absorption in the colon, butyrate serves as the main energy source for colonocytes, while concurrently enhancing epithelial barrier integrity and exhibiting notable anti-inflammatory effects [20]. Among the recognized butyrate-producing bacteria, *Clostridium butyricum* (*C. butyricum*) has emerged as the most clinically relevant probiotic for alleviating symptoms associated with gastrointestinal infections [21]. Experimental models of CDI have demonstrated that administration of *C. butyricum* effectively reduces both the incidence of diarrhea and mortality rates [22]. Moreover, *C. butyricum* metabolites exhibit targeted activity against *C. difficile*-induced dysbiosis through dual mechanisms of toxin suppression and growth inhibition, coupled with restoration of intestinal homeostasis [23].

Nevertheless, existing research does not adequately evaluate the functional effects of natural bioactive compounds sourced from plants and microbes, respectively, on CDI, particularly in terms of their influence on host-microbe interactions and the associated regulatory mechanisms. This deficiency in knowledge substantially impedes the advancement of sophisticated anti-*C. difficile* strategies that utilize natural products and probiotics.

Mass spectrometry-based proteomics has emerged as a formidable methodology for elucidating molecular mechanisms across a spectrum of diseases, including infectious diseases [24, 25]. Within microbial pathogenesis, it facilitates the comprehensive characterization of host–pathogen interactions and the identification of antimicrobial targets [26]. Proteomic methodologies offer distinct advantages in investigating microbial post-translational modifications (PTMs), yielding novel insights into pathogen virulence. For instance, in *Neisseria meningitidis*, top-down proteomic analysis of the type IV pilin PilE revealed a rapid upregulation of glycerylphosphorylation within hours of host contact, identifying this PTM as a molecular switch that regulates bacterial dissemination and invasive infection [27]. Recent research has demonstrated that natural bioactive compounds can exert antimicrobial effects by specifically targeting microbial PTMs. For instance, a multi-omics dissection of *Ginkgo biloba* recently revealed that lysine acetylation (Kac) dynamically regulates the entire flavonoid-biosynthetic pathway; the pharmacological blockade of deacetylases rapidly amplifies the accumulation of antibacterial flavonoids, illustrating a plant-based strategy for PTM-directed chemical intervention [28]. Beyond direct host–pathogen interactions, microbiota-derived SCFAs act as endogenous HDAC inhibitors that elevate global Kac within colonocytes, thereby reprogramming metabolic and cell-cycle programs and fortifying the epithelial barrier against enteric infection [29]. Building on these findings, the present study utilizes proteomic methodologies to explore how natural compounds derived from plants and microbes modulate PTMs in *C. difficile* to achieve antibacterial effects.

In this study, we systematically investigate the mechanisms by which the plant flavonoid XN and the probiotic metabolite preparation *C. butyricum* supernatant (CBs) counteract *C. difficile* colonization and infection. These two agents are examined in parallel as independent interventions, each evaluated for its ability to reduce bacterial colonization, inhibit pathogen growth, and limit toxin production. Using a dextran sulfate sodium (DSS)-induced Caco-2 intestinal epithelial barrier model, we demonstrated that XN effectively preserves epithelial barrier integrity, mitigates *C. difficile*-induced damage, and downregulates associated inflammatory signaling pathways. High-resolution mass spectrometry reveals that these two approaches, XN and CBs, both induce significant alterations in the pathogen's proteomic profile and broadly modulate lysine acetylation. Integrated bioinformatics analysis shows that each intervention influences glycolysis pathways via acetylation-dependent regulation of the key metabolic enzyme fructose-1,6-bisphosphate aldolase (FBA). These findings support the development of PTM-targeted strategies against bacterial pathogenesis and highlight glycolysis modulation as a promising therapeutic avenue for CDI. By examining XN and CBs as distinct yet parallel models, this work elucidates their shared mechanistic basis in targeting the lysine acetylation network of *C. difficile* and provides novel proteomic insights into their antibacterial actions.

## Materials and methods

### Bacterial strains culture

*C. difficile* strain ATCC BAA-1382 and *C. butyricum* strain ATCC 19398 were obtained from the BeNa Culture Collection. As established in the literature [30, 31], both *C. difficile* and *C. butyricum*, as commensal inhabitants of the human gut, are strictly anaerobic microorganisms. *C. difficile* was cultured anaerobically at 37 °C in brain heart infusion broth (Haibo, HB8297) supplemented with 0.5% yeast extract (OXOID, LP0021B) and 0.1% L-cysteine (Sigma-Aldrich, 102838) under anaerobic conditions until reaching the logarithmic growth phase. *C. butyricum* was cultured anaerobically in bifidus selective medium (Haibo, HB0394-1) supplemented with 0.25% L-cysteine at 37 °C for 12 h until reaching the stationary phase under the same anaerobic conditions. The cell-free supernatant, designated as CBs, was collected by centrifugation at 8000 rpm for 5 min and subsequently sterilized by passage through a 0.22 μm pore size membrane filter. After a 24 h treatment of *C. difficile* with either XN (Felix, F423095) or CBs, the bacterial cells were harvested by centrifugation at 8000 rpm for 5 min. The pellets were subsequently washed twice with phosphate-buffered saline (PBS, Servicebio) to remove residual medium components, followed by protein extraction for subsequent proteomic analysis.

### Caco-2 cell culture

The human colorectal adenocarcinoma cell line Caco-2 (Servicebio) was maintained in Dulbecco’s modified Eagle’s medium (DMEM, Servicebio, G4516) supplemented with 10% fetal bovine serum (FBS, Servicebio, G8003) and 1% penicillin/streptomycin. Cells were cultured at 37 °C in a humidified atmosphere of 5% CO_2_ / 95% air and passaged at 70–90% confluence. For the Caco-2 colonization assay, cells were seeded in a 96-well plate at 1 × 10^5^ cells per well and cultured for 24 h before being subjected to the assay. Separately, for the assessment of DSS-induced injury on cell viability, cells were similarly seeded and cultured for 24 h, then incubated with 3% DSS for an additional 24 h to induce IBD-like injury, after which cell viability was measured using the Cell Counting Kit-8 assay [32, 33].

### Caco-2 colonization assay

Following 24 h of incubation, the medium was replaced with complete medium containing XN or CBs and incubated for an additional 3 h prior to the colonization assays, according to the reported research method [33]. *C. difficile* cultures in logarithmic growth phase were harvested by centrifugation at 8,000 rpm for 5 min, washed twice with PBS, and resuspended in PBS containing 5(6)-carboxyfluorescein diacetate succinimidyl ester (MCE, HY-D0938). After 30 min of anaerobic incubation at 37 °C with 220 rpm shaking in the dark, the reaction was terminated by adding DMEM with 40% FBS. Bacterial suspensions were adjusted to 1 × 10⁸ CFU/mL in PBS and added to Caco-2 monolayers for 3 h coculture at 37 °C. Unattached bacteria were removed by three gentle PBS washes, and fluorescence intensity was measured at 485/538 nm (excitation/emission) using a Synergy H1 multimode microplate reader (BioTek).

### Time-point bacterial growth assay

*C. difficile* was cultured anaerobically to the logarithmic growth phase as described in the “Bacterial strains culture” section. The cultures were then supplemented with either XN at a final concentration of 50 μM or CBs at a CBs:*C. difficile* ratio of 1:100 and continued to be incubated under anaerobic conditions. Bacterial proliferation was assessed by measuring the optical density at 600 nm (OD_600_) after 12 h of culture.

### Cell counting kit-8 assay

After 48 h culture, 10% (v/v) CCK-8 solution was added to the cells in the 96-well plate, followed by incubation at 37 °C for 2 h. Absorbance was measured at 450 nm.

### Quantitative real-time PCR analysis

Bacterial cultures were grown as described in the “Bacterial strains culture” section, and cells were harvested by centrifugation for total RNA isolation using the Trizol method. To ensure genomic DNA removal, RNA samples were treated with HiScript III RT SuperMix for qPCR (+ gDNA wiper) (Vazyme, R323-01), followed by reverse transcription to generate cDNA. qRT-PCR was performed on a QuantStudio 5 Real-Time PCR System (Thermo Fisher, A28569) using SYBR Green Master Mix (Servicebio, G3326). Gene expression levels of t*cdA*, *tcdB*, and *tcdR* were normalized to the internal control *rpoB* using the 2^−ΔΔCT^ method. The primer sequences used in this study are provided in Table S6.

### Statistical analysis

All experiments were performed in triplicate unless otherwise stated. Data were analyzed using GraphPad Prism 9.5. The specific statistical tests applied were chosen based on the experimental design. For comparisons between two groups only, data normality was first assessed using Normality and Lognormality Tests, followed by two-tailed Student’s t-tests. For comparisons involving three or more groups, one-way ANOVA was performed, followed by Tukey’s post hoc test for multiple comparisons. In all analyses, a p-value of < 0.05 was considered statistically significant.

### Protein extraction

Bacterial cultures were grown as described in the “Bacterial strains culture” section, bacterial cells were lysed in ice-cold buffer containing 100 mM NH_4_HCO_3_, 8 M urea, protease inhibitors, and phosphatase inhibitors for 30 min. The lysate was subsequently sonicated for 5 min and centrifuged at 15,000 rpm for 10 min to collect the supernatant, protein concentration was determined using a BCA assay kit (Beyotime, P0011).

### In-Solution trypsin digestion

Protein samples were reduced with 5 mM dithiothreitol at 56 °C for 30 min to cleave disulfide bonds, followed by alkylation with 15 mM iodoacetamide in the dark at room temperature for 30 min. The alkylation reaction was terminated with 30 mM cysteine for 30 min. Then, trypsin was added at an enzyme-to-protein ratio of 1:50 (w/w), and the digestion was carried out at 37 °C for 16 h. A second digestion was then performed with trypsin at a ratio of 1:100 (w/w) for an additional 4 h. Finally, the digested peptides were desalted using a Sep-Pak C18 cartridges (Waters).

### TMT labeling

The dried peptides were dissolved in 100 mM TEAB buffer (Sigma Aldrich; 15715-58-9) at a final concentration of 5 μg/μL and pH 8.0 and then labeled with tandem mass tag (TMT) reagents (Thermo Fisher, #YL379915) at a peptide-to-TMT ratio of 1:2 (w/w). After vortexing and centrifugation, the reaction was carried out at 25 °C and 900 rpm for 1 h. Following the labeling reaction, a small aliquot was analyzed to verify labeling efficiency, with the requirement that ≥ 95% of both N-terminal and lysine (K) side-chain amines were modified. Once the standard was met, the remaining samples were quenched and pooled in equimolar amounts. Finally, the labeled peptides were desalted using Sep-Pak C18 cartridges.

### Affinity enrichment of acetylated peptides

The acetylated peptide enrichment was conducted following our previously established protocols [34]. The peptide samples were dissolved in ETN buffer (1.5 M Tris–HCl, 100 mM EDTA-2Na, 5 M NaCl, pH 8.0) and incubated overnight at 4 °C with PBS-prewashed acetylated antibody beads (Immunechem; ICP0388) with gentle shaking. The following day, the beads were sequentially washed with NETN buffer (0.5% NP-40, 50 mM Tris–HCl, 1 mM EDTA-2Na, 600 mM NaCl, pH 8.0), ETN buffer and ddH₂O. Bound peptides were eluted twice with 0.1% trifluoroacetic acid (TFA), followed by two additional elutions using 30% acetonitrile (ACN) in 0.1% TFA. The eluted peptides were then vacuum-dried and desalted using C18 ZipTips (Merck, ZTC18S) prior to liquid chromatography-tandem mass spectrometry (LC–MS/MS) analysis. BioRender (http://biorender.com/) was used to create the workflow and graphical abstract.

### LC–MS/MS analysis

For unenriched peptide samples, desalted peptides were dissolved in solvent A (0.1% formic acid, 2% acetonitrile in water, v/v) and analyzed using a Vanquish Neo UHPLC system (Thermo Fisher Scientific) coupled to an Orbitrap Ascend mass spectrometer equipped with a DNV75150 PN-C18 column (Thermo Fisher Scientific; 75 μm inner diameter × 18 cm length, 2 μm C18 particles). Peptides were separated through a 25 min gradient program, starting from 4 to 5% solvent B (0.1% formic acid in acetonitrile, v/v) for 2 min, then from 5% to 22.5% solvent B for 14 min, from 22.5% to 35% solvent B for 5 min, and from 35 to 55% solvent B for 0.5 min. Finally, the column was washed with 99% solvent B for 3.5 min at a constant flow rate of 700 nL/min. The eluted peptides were ionized by electrospray and full scans were performed in the Orbitrap Ascend mass spectrometer within the m/z range of 380–980, with a resolution of 120,000 at m/z 200 and a maximum injection time of 50 ms. HCD fragmentation with 25% normalized collision energy was applied to peptides with 2–6 charge states, and fragment ions were detected in the ion trap with an MS2 AGC target of 30,000.

For lysine-acetylated peptide analysis, the same platform was employed with modified parameters. Peptides were separated through a 110 min gradient program, starting from 5 to 7% solvent B for 0.5 min, then from 7 to 30% solvent B for 97.5 min, and from 30 to 50% solvent B for 5 min. Finally, the column was washed with 99% solvent B for 7 min at a flow rate of 500 nL/min. The mass spectrometry scan range was extended to 400–1600 m/z maintaining a resolution of 120,000 at m/z 200 and a maximum injection time of 50 ms. HCD collision energy was increased to 35% (for 2–6 charge state peptides), and the MS2 AGC target was elevated to 150,000 to enhance detection sensitivity for low-abundance modified peptides.

### Mass spectrometry data processing

The raw MS data were processed using MaxQuant software (version 2.4.14.0) with reference to the UniProt database (Proteome ID: UP000001978, containing 3762 protein entries) for *C. difficile*. The search parameters were configured as follows: Trypsin/P was specified as the protease with a maximum of two missed cleavages permitted; up to three variable modifications were allowed. Carbamidomethylation of cysteine (C) was set as the fixed modification, while variable modifications included methionine oxidation (M), protein N-terminal acetylation, and lysine acetylation (Kac). To ensure statistical rigor, both protein and peptide identifications were filtered at a false discovery rate (FDR) threshold of ≤ 1% (FDR ≤ 0.01).

### Bioinformatics analysis

During data processing, proteins identified as contaminants or reverse sequences were systematically excluded. Only peptides with a modification site localization probability > 0.75 were retained for subsequent analyses. Differential expression thresholds were set according to data type. For protein expression profiling, differentially expressed proteins (DEPs) were defined using a FC > 1.5 and a p-value < 0.05, a threshold selected to capture biologically meaningful changes in protein abundance while ensuring stringency. In the acetyl-proteomics analysis, a more sensitive cutoff of FC > 1.2 (p < 0.05) was applied, reflecting the typically subtler yet functionally significant quantitative shifts associated with Kac. Pathway enrichment and protein–protein interaction (PPI) network construction were performed using the STRING database (version 12.0), with the confidence threshold set to 0.7. Furthermore, key functional modules within the PPI network were identified through cluster analysis using Cytoscape software (version 3.9.1) and its molecular complex detection (MCODE) plugin.

### Plasmid construction and protein purification

For protein purification of FBA, we constructed recombinant plasmids pET28a-FBA and pET28a-FBA K280A using the pET28a expression vector. All constructs were sequence-verified prior to use. The verified plasmids were individually transformed into *E. coli* BL21 competent cells (Tsingke Biotechnology, TSC-C14) and cultured in Lysogeny Broth (LB) liquid medium supplemented with 0.05 M kanamycin at 37 °C with 220 rpm shaking for 16 h. When the culture OD_600_ value reached 0.4–0.6, protein expression was induced by adding 0.1 mM isopropyl-β-D-thiogalactopyranoside (IPTG), followed by incubation at 16 °C with 160 rpm shaking for 16 h. The bacterial cells were harvested by centrifugation at 8500 rpm for 5 min at 4 °C. After discarding the supernatant, the cell pellet was resuspended in 10 mM imidazole PBS buffer and thoroughly vortexed. The cell suspension was then sonicated for 5 min (three times) and centrifuged at 8500 rpm for 20 min. The resulting supernatant was subjected to Ni–NTA agarose resin affinity chromatography for target protein purification.

### Enzyme assay

The activity of fructose-1,6-bisphosphate aldolase was measured using a previously reported method [35]. The assay employed a coupled enzymatic system consisting of triosephosphate isomerase and glyceraldehyde-3-phosphate dehydrogenase. The reaction mixture (250 μL) contained 50 mM Tris–HCl (pH 7.2), 0.1 mM fructose-1,6-bisphosphate (FBP), 0.15 mM NADH, 1 mM EDTA, 0.2 mM EGTA, 10 U/mL triosephosphate isomerase, and 1 U/mL glyceraldehyde-3-phosphate dehydrogenase. Reactions were initiated at 30 °C, and enzyme activity was quantified by monitoring the oxidation rate of NADH at 340 nm.

## Results

### *C. difficile* exacerbates DSS-induced injury in Caco-2 cells, while XN mitigates this effect

**Fig. 1:**
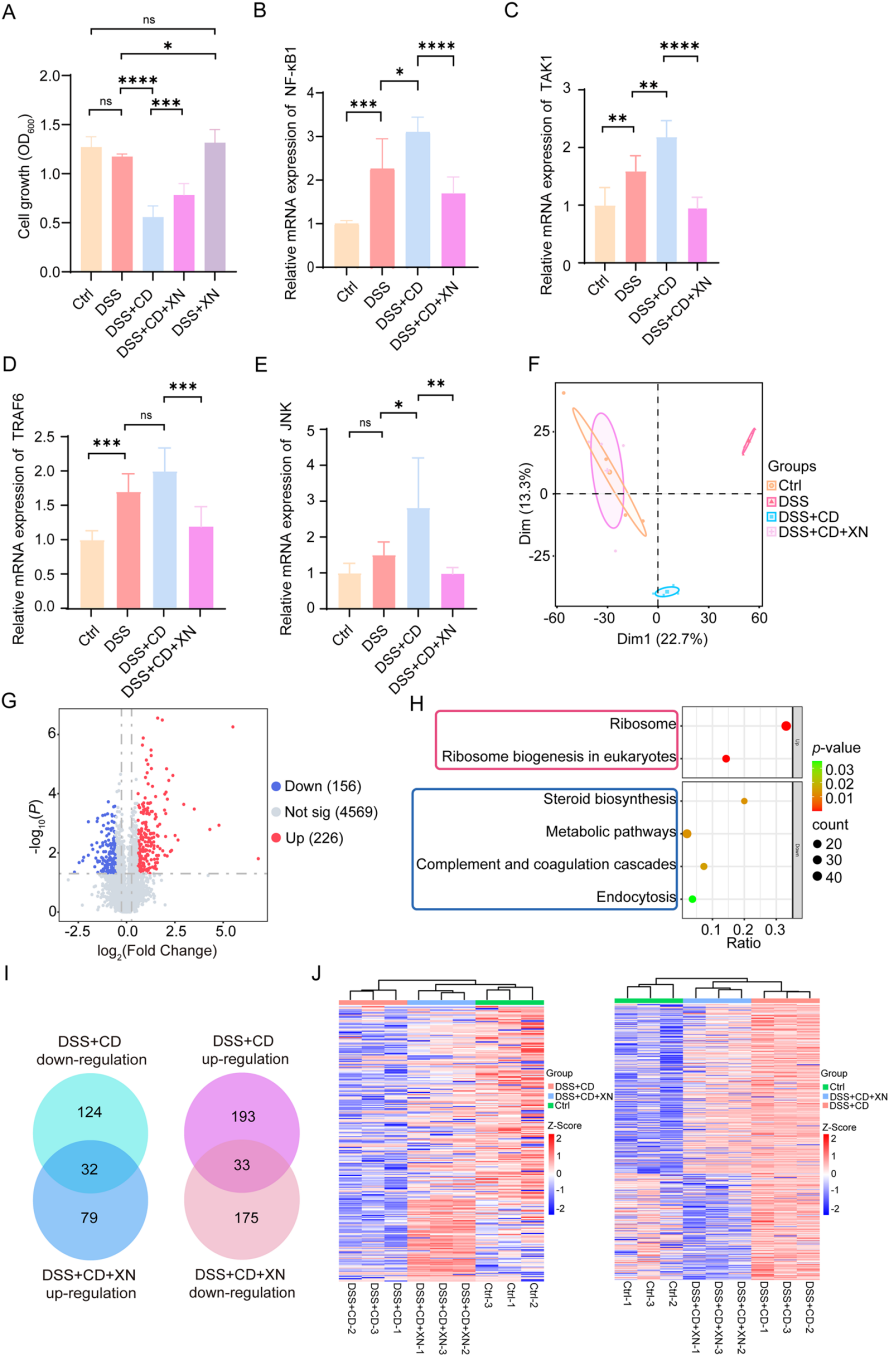
*C. difficile* exacerbates DSS-induced injury in Caco-2 cells, while XN ameliorates this effect. **A** Cell viability assessed by Cell Counting Kit-8 assay. **B**–**E** Expression levels of inflammatory pathway components (NF-κB1, TAK1, TRAF6, and JNK) quantified by qRT-PCR. **F** Principal component analysis (PCA) of the Caco-2 cell proteome. **G** Volcano plot of DEPs in Caco-2 cells following DSS induction and CDI versus normal control cells (*p* < 0.05, fold change (FC) > 1.5). **H** KEGG pathway enrichment analysis of DEPs in DSS-induced and CDI Caco-2 cells versus normal control cells. **I** Venn diagram comparing DEPs between *C. difficile* and XN interventions. **J** Heatmap of Caco-2 cell proteome dynamics (left heatmap): proteins suppressed by CDI and restored by XN treatment; (right heatmap): proteins upregulated upon CDI and downregulated by XN intervention. Data represent mean ± SD (ns, *p* > 0.05; *, *p* < 0.05; **, *p* < 0.01; ***, *p* < 0.001****, *p* < 0.0001)

Following established experimental protocols [36], we initially induced inflammatory injury in human colorectal adenocarcinoma Caco-2 cells using DSS. To investigate host-microbe interactions, we subsequently assessed cellular viability after exposure to *C. difficile*. Our results indicate that DSS alone significantly reduced cell survival rates, and concurrent infection with *C. difficile* exacerbated this cytotoxic effect (Fig. 1A). Notably, administration of XN substantially mitigated the reduction in viability. The inflammatory signaling pathways activated by host-bacterial interactions are critical determinants of CDI pathogenesis. As shown in Fig. 1B–E and Fig. S1A, DSS increased the expression levels of key inflammatory pathways, including NF-κB1, TAK1, TRAF6, and JNK, in Caco-2 cells. Subsequent infection with *C. difficile* further enhanced this upregulation. Importantly, XN consistently downregulated the expression of all four pathways, underscoring its significant anti-inflammatory properties.

Using the quantitative proteomic analysis, we conducted a comprehensive evaluation of the effects induced by XN. PCA revealed the overall distribution and intergroup separation trends of the proteomic data among the experimental groups. To quantitatively assess the intergroup differences, a PERMANOVA was performed, which confirmed that the experimental grouping had a significant effect on the proteomic variation (p = 0.001, R^2^ = 0.668). This indicates that both DSS and *C. difficile* treatments elicited significant alterations in the proteomic profiles of Caco-2 cells (Fig. 1F, Table S1). Notably, in the PCA, the samples from the XN-treated group clustered closely with those from the untreated control group. This pattern aligns with the observations from the qRT-PCR results. The spatial proximity in the PCA plot suggests an association between XN treatment and the proteomic perturbations induced by CDI. Additionally, our analysis identified significantly altered proteins based on FC and reproducibility assessments, delineating 156 and 226 differentially expressed genes in the *C. difficile* group, respectively (Fig. 1G). Recognizing the pivotal role of energy metabolism in host–pathogen interactions, the Kyoto Encyclopedia of Genes and Genomes (KEGG) was utilized to categorize these genes into functional groups. Our study demonstrated that CDI significantly downregulated proteins involved in steroid biosynthesis (Fig. 1H, Fig. S1B, C). Given the established role of sterol metabolites in maintaining epithelial membrane integrity and tight junction function [37–39], this downregulation suggests a potential metabolic vulnerability that could compromise barrier defense and facilitate pathogen colonization and toxin action.

Through comparative proteomic analysis between the *C. difficile* and XN groups, we identified 32 proteins that were downregulated by *C. difficile* but restored by XN, as well as 33 proteins exhibiting inverse regulatory patterns (Fig. 1I). Hierarchical clustering analysis using Euclidean distance revealed an antagonistic regulatory pattern between the *C. difficile* and XN treatment groups. This analysis provided a global perspective on the proteomic alteration trends and offered a visual representation of the differences among treatment groups at the molecular level (Fig. 1J). The profound alterations in the host proteome indicate a fundamental rewiring of cellular processes under the combined stress of DSS and CDI. Crucially, our data reveal a targeted antagonistic relationship between XN and the pathogen, as XN counteracts the proteomic signature induced by *C. difficile* and restores the expression of proteins. Collectively, these proteomic findings support the hypothesis that CDI amplifies DSS-induced epithelial injury in Caco-2 cells, while XN confers protection against this synergistic damage.

### XN suppresses *C. difficile* progression and induces global proteomic remodeling

**Fig. 2:**
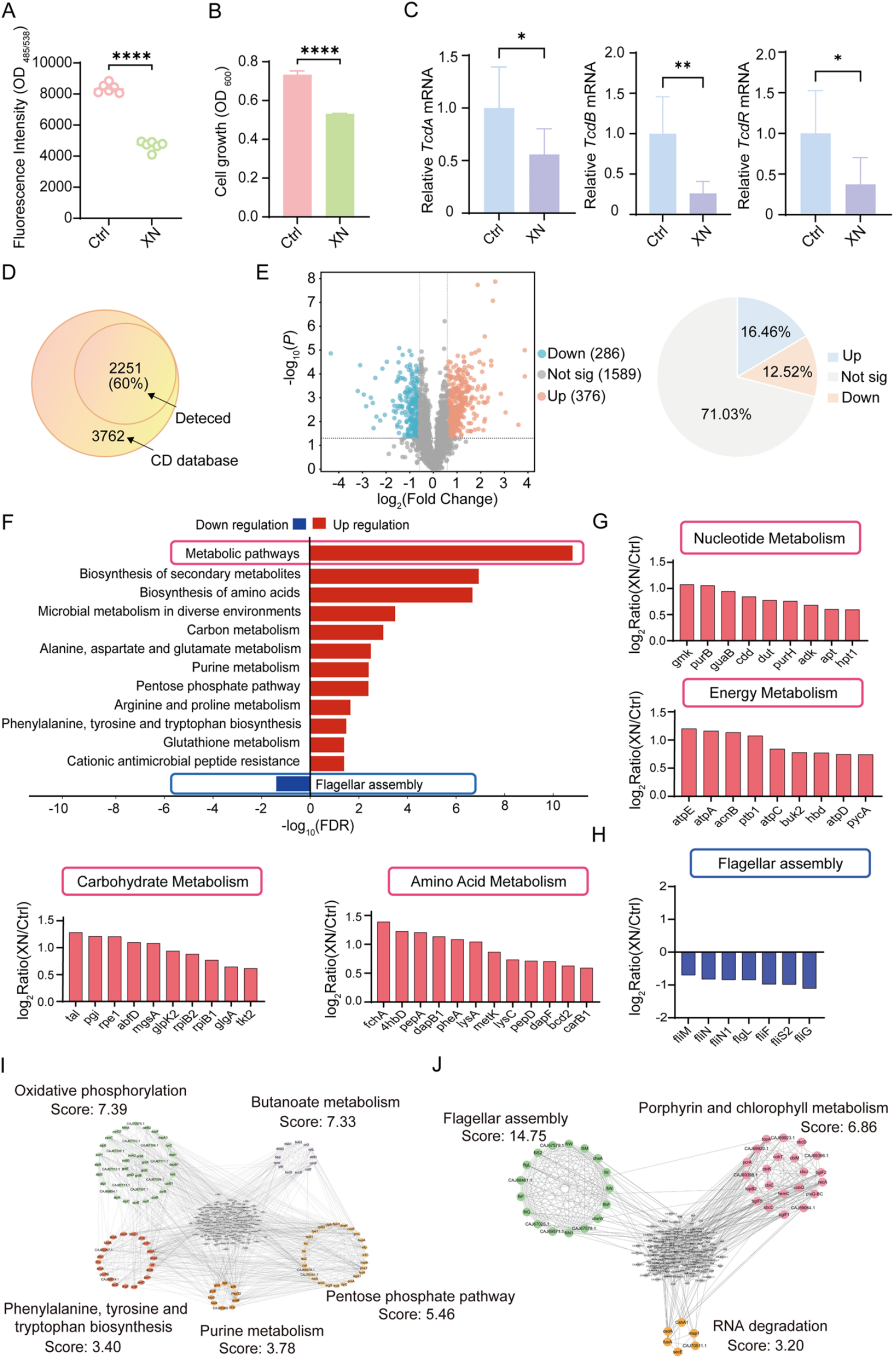
XN suppresses *C. difficile* progression and orchestrates global proteomic remodeling. **A** Colonization of carboxyfluorescein succinimidyl ester (CFSE)-labeled *C. difficile* to Caco-2 cells, measured by fluorescence intensity. **B** Bacterial proliferation assay of *C. difficile*. **C** Transcriptional analysis of *C. difficile* toxin-encoding genes (t*cdA*, t*cdB*, and t*cdR*). **D** Total number of proteins identified in *C. difficile*. **E** Volcano plot and proportional distribution of DEPs (*p* < 0.05, FC > 1.5). **F** KEGG pathway analysis of DEPs. **G** Bar plot showing the upregulated proteins identified in the metabolic pathway. **H** Bar plot showing the downregulated proteins identified in the flagellar assembly. **I**, **J** PPI networks of upregulated proteins (**I**) and downregulated proteins (**J**). Data represent mean ± SD (ns, *p* > 0.05; *, *p* < 0.05; **, *p* < 0.01; ***, *p* < 0.001; ****, *p* < 0.0001; Student’s t-test)

To systematically assess the inhibitory effects of XN on *C. difficile* interaction with host cells, we employed CFSE fluorescence labeling to quantify total cell-associated bacteria, which includes both adherent and potentially internalized bacteria (Fig. 2A), suggesting a potential protective role of XN through the inhibition of pathogenic colonization. Bacterial growth kinetic assays further corroborated the direct suppressive effect of XN on *C. difficile* proliferation (Fig. 2B). Considering the established roles of the toxins *tcdA* and *tcdB*, along with their transcriptional regulator *tcdR*, in mediating colonic inflammation and epithelial damage-hallmark pathological features of symptomatic CDI [40], we conducted a quantitative analysis of toxin gene expression. Our findings demonstrated that XN treatment significantly downregulated the transcript levels of *tcdA*, *tcdB*, and t*cdR* (Fig. 2C, Fig. S1D). This transcriptional reduction is indicative of an inhibition of toxin gene expression, which may result from the global impact of XN on bacterial physiology. These collective findings demonstrate that XN exerts multifaceted regulation over the pathogenicity of *C. difficile* by simultaneously modulating colonization, proliferation, and toxin production. Building on these insights, we conducted further investigations to elucidate the antimicrobial mechanism of XN. By supplementing *C. difficile* cultures with XN and the vehicle solvent for comprehensive proteomic analysis, we characterized the dynamic proteomic changes in *C. difficile* within the XN-treated group, identifying 2251 proteins. This represents 60% coverage of the known proteome (3762 entries) for this species in the UniProt database, demonstrating the robustness of our dataset (Fig. 2D, Table S2). Comparative analysis identified 662 DEPs (286 downregulated and 376 upregulated), accounting for 29% of the total detected proteins (Fig. 2E). These substantial modifications indicate that XN may disrupt the physiology of *C. difficile* by influencing multiple biological processes. To systematically explore the functional ramifications of these proteomic alterations, we performed a KEGG pathway enrichment analysis, which revealed 13 significantly altered pathways. Notably, pathways related to amino acid biosynthesis, secondary metabolite production, and carbon metabolism were the most prominently affected (Fig. 2F). Further characterization of the biological processes and subcellular localization indicated that these proteomic changes predominantly occur within the cytoplasmic compartment (Fig. S2A), with significant involvement in small molecule metabolism, organic acid processing, and carboxylic acid metabolic pathways (Fig. S2B). These findings collectively indicate that XN induces significant metabolic disruptions in *C. difficile*. A detailed analysis of the DEPs within the most significantly enriched pathways revealed an upregulation of nearly all enzymatic components associated with four fundamental metabolic processes: nucleotide metabolism, amino acid metabolism, carbohydrate metabolism, and energy production (Fig. 2G). This pattern suggests that *C. difficile* may engage in extensive metabolic reprogramming through the global activation of these pathways as a compensatory response to XN-induced stress. Conversely, we observed a pronounced downregulation of proteins involved in flagellar assembly (Fig. 2H), which is consistent with our previous findings of XN-induced inhibition of *C. difficile* colonization to intestinal epithelial cells.

To systematically characterize the interaction networks of DEPs, we performed an extensive network analysis using high-confidence interaction data obtained from the STRING database [41]. Network topology analysis revealed that upregulated proteins formed highly interconnected modules predominantly associated with oxidative phosphorylation pathways (Fig. 2I). Whereas downregulated proteins were significantly enriched in components related to flagellar assembly (Fig. 2J). The observed downregulation of flagellar assembly components in the proteomic data offers a potential molecular explanation for the impaired colonization of *C. difficile* following XN treatment, as demonstrated in our cellular assays.

### CBs attenuates *C. difficile* pathogenesis and reprograms the proteomic landscape

**Fig. 3:**
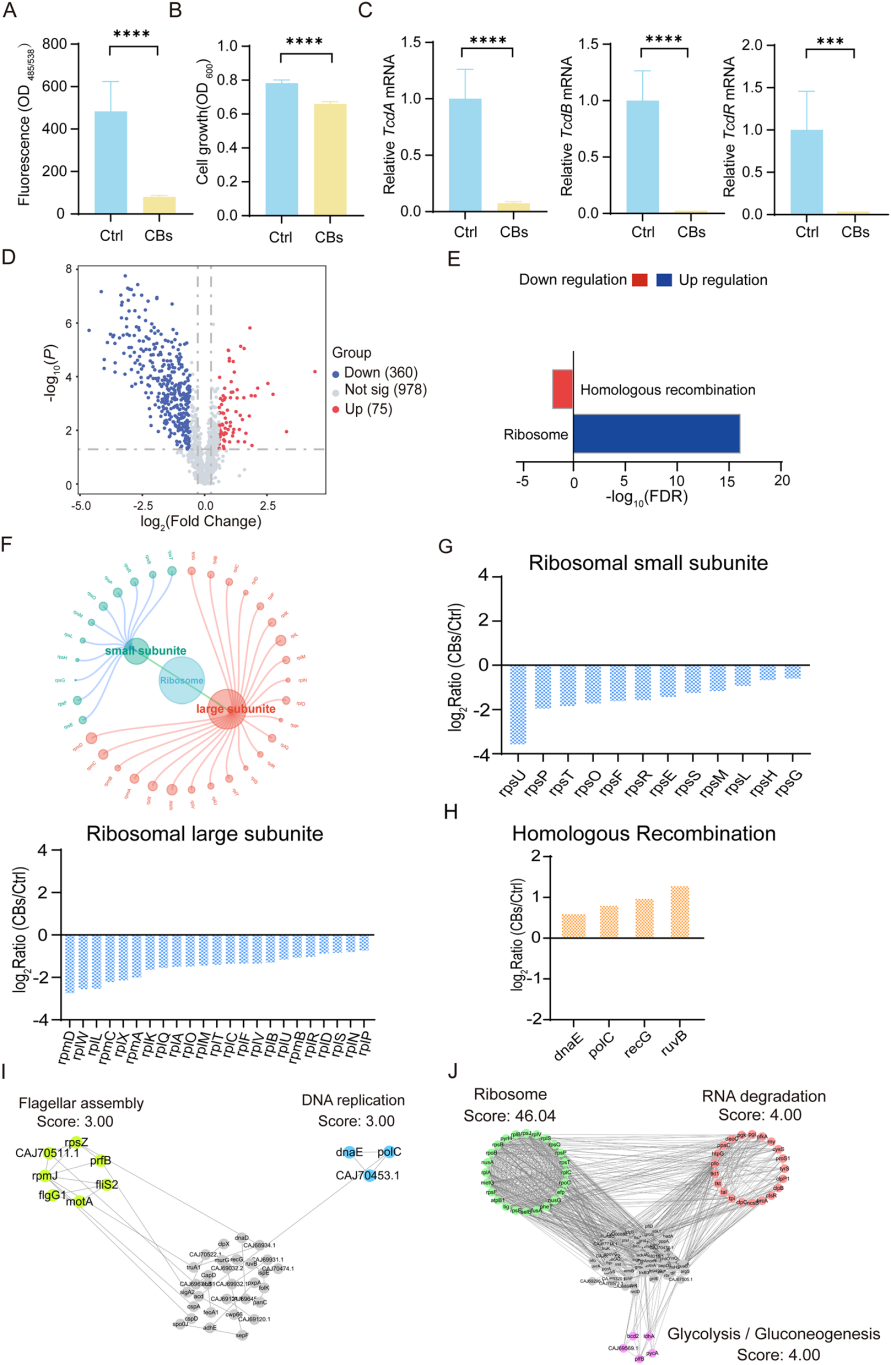
CBs attenuates *C. difficile* pathogenesis and reshapes the proteomic landscape. **A** Colonization of CFSE-labeled *C. difficile* to Caco-2 cells following CBs intervention. **B** Growth dynamics of *C. difficile* exposed to CBs. **C** qRT-PCR analysis of *C. difficile* toxin genes (t*cdA*, t*cdB*, and t*cdR*). **D** Volcano plot of DEPs (*p* < 0.05, FC > 1.5). **E** KEGG pathway enrichment. **F** Hierarchical network of downregulated ribosomal pathways. **G** Bar plots highlighting the upregulated proteins identified in the homologous recombination pathway. **H** Bar plots highlighting the downregulated proteins identified in the ribosomal pathway. **I**, **J** PPI networks of upregulated proteins (**I**) and downregulated proteins (**J**). Data represent mean ± SD (ns, *p* > 0.05; *, *p* < 0.05; **, *p* < 0.01; ***, *p* < 0.001; ****, *p* < 0.0001; Student’s t-test)

Given the observed efficacy of XN, we next sought to investigate the anti-CDI potential of CBs, a microbially-derived metabolite preparation, using an analogous experimental workflow to evaluate its effects as a parallel therapeutic candidate. Our experimental findings indicate that the CBs group, in alignment with the previously documented effects of XN, significantly reduced the colonization capacity of *C. difficile*, inhibited bacterial proliferation, and suppressed toxin production (Fig. 3A–C, Fig. S3). These results confirm that CBs exhibit anti-*C. difficile* activity comparable to that of XN. Proteomic analysis revealed that CBs induced substantial proteome remodeling in *C. difficile*, characterized by the downregulation of 360 proteins and the upregulation of 75 proteins (Fig. 3D, Table S3). KEGG pathway enrichment analysis suggested that these DEPs are predominantly involved in two essential biological processes: homologous recombination and ribosome biogenesis (Fig. 3E).

Through network analysis, further characterization of downregulated ribosomal protein expression patterns revealed that 23 proteins from the large subunit and 12 proteins from the small subunit were significantly downregulated (Fig. 3F). This coordinated reduction in components of the protein synthesis machinery (Fig. 3G) indicates a substantial impairment of translational capacity, potentially restricting the proliferation of *C. difficile*. Conversely, we observed an upregulation of four key proteins involved in homologous recombination repair (Fig. 3H). PPI network analysis further revealed a close association between these protein clusters and the glycolysis/gluconeogenesis and flagellar assembly pathways (Fig. 3I, J). Notably, both XN and CBs induced proteomic modifications in identical components of the flagellar assembly, leading us to hypothesize that these compounds may target *C. difficile* through a shared critical regulatory mechanism.

### Systems-level profiling of lysine acetylation in *C. difficile* in response to XN

**Fig. 4:**
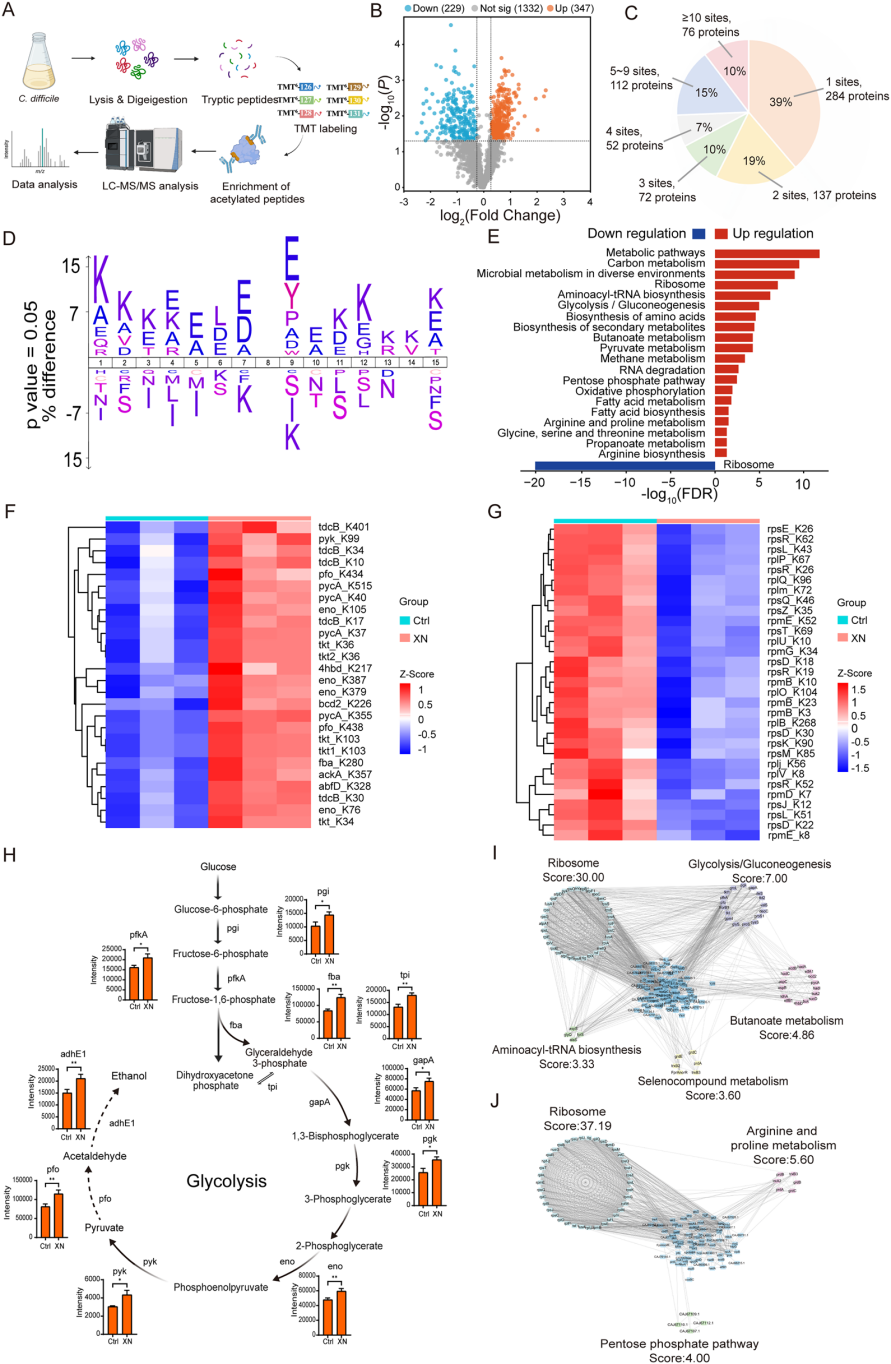
Systems-level profiling of Kac in *C. difficile* in response to XN. **A** Schematic workflow for acetylome analysis. **B** Volcano plot of acetylated peptides (*p* < 0.05, FC > 1.2). **C** Distribution of 2653 acetylation sites across 733 proteins. **D** IceLogo analysis of amino acid motifs flanking acetylation sites. **E** KEGG pathway mapping of acetylated proteins. **F** Heatmap of lysine-acetylated proteins upregulated in carbon metabolism. **G** Heatmap of lysine-acetylated proteins downregulated in ribosomal pathways. **H** Glycolysis pathway annotated with identified enzymes: pgi, glucose-6-phosphate isomerase; fba, fructose-1,6-bisphosphate aldolase; tpi, triosephosphate isomerase; gapA, glyceraldehyde-3-phosphate dehydrogenase; pgk, phosphoglycerate kinase; eno, enolase; pyk, pyruvate kinase; pfo, pyruvate ferredoxin oxidoreductase; adhE1, aldehyde-alcohol dehydrogenase; pfkA, ATP-dependent 6-phosphofructokinase. **I**, **J** PPI networks of proteins with increased (**I**) or decreased (**J**) acetylation levels. Data represent mean ± SD (ns, *p* > 0.05; *, *p* < 0.05; **, *p* < 0.01; ***, *p* < 0.001; ****, *p* < 0.0001; Student’s t-test)

Based on the proteomic expression profiles, we found that the levels of several key acetyltransferases and deacetylases were significantly altered following XN and CBs interventions (Fig. S4). To gain deeper mechanistic insights into XN-induced molecular alterations in *C. difficile*, we performed comprehensive analysis of lysine acetylome. Utilizing a comprehensive TMT-based quantitative acetyl-proteomics approach, we systematically delineated the Kac profile in *C. difficile* following interventions with XN (Fig. 4A). This analysis identified 576 proteins with significant alterations in expression levels, comprising 229 downregulated and 347 upregulated proteins in response to XN treatment (Fig. 4B, Table S4). We successfully identified 2653 Kac sites across 733 proteins, thereby significantly expanding the catalog of known acetylation sites and substrates in *C. difficile*. An assessment of the distribution of acetylation sites revealed that 61% of the identified proteins harbored multiple modification sites (Fig. 4C), underscoring the extensive role of Kac in the regulation of the *C. difficile* proteome.

To further elucidate the specificity of lysine modification, we conducted an examination of amino acid preferences in the vicinity of modified lysine residues. Utilizing IceLogo software, we employed a statistical sequence background derived from all lysine-containing peptides in the UniProt *C. difficile* database to analyze the flanking sequences of acetylation sites and assess alterations in amino acid preferences of lysine substrates. The analysis indicated a predominant occurrence of glutamate residues at the + 1 position, whereas lysine residues were significantly more prevalent than glutamate at the + 4 position (Fig. 4D). Additionally, we mapped acetylated proteins to KEGG metabolic pathways to investigate the acetylation of metabolic enzymes. The findings revealed that a majority of metabolic enzymes associated with carbon metabolism, microbial metabolism in diverse environments, and glycolysis/gluconeogenesis pathways were acetylated (Fig. 4E).

In addition, we conducted a systematic analysis of DEPs within significantly enriched pathways, which revealed distinct Kac patterns across various functional protein categories. As illustrated in Fig. 4F, pathways involved in carbon metabolism exhibited significant upregulation, with glycolysis enzymes, such as fba_K280 and eno_K105, showing notable hyperacetylation. This finding suggests that XN induces metabolic reprogramming to enhance energy production capacity. Conversely, ribosomal proteins demonstrated significant downregulation (Fig. 4G), indicating that XN preferentially targets the translation machinery. Given that glycolysis is a highly representative pathway, we further characterized its metabolic flux and quantified the abundance of identified metabolic enzymes. These enzymes, which essentially encompass the entire glycolysis process, showed increased abundance (Fig. 4H). To elucidate the global regulatory role of Kac in the metabolism of *C. difficile*, we developed PPI networks utilizing the STRING database, applying stringent interaction confidence thresholds (score > 0.7). Subsequent network analysis was conducted using Cytoscape software in conjunction with the MCODE plugin to identify highly interconnected functional modules. The analysis of the resulting networks indicated extensive acetylation of proteins involved in essential metabolic pathways, notably glycolysis/gluconeogenesis and arginine/proline metabolism (Fig. 4I, J).

### Global acetylome profiling of *C. difficile* in response to CBs

**Fig. 5:**
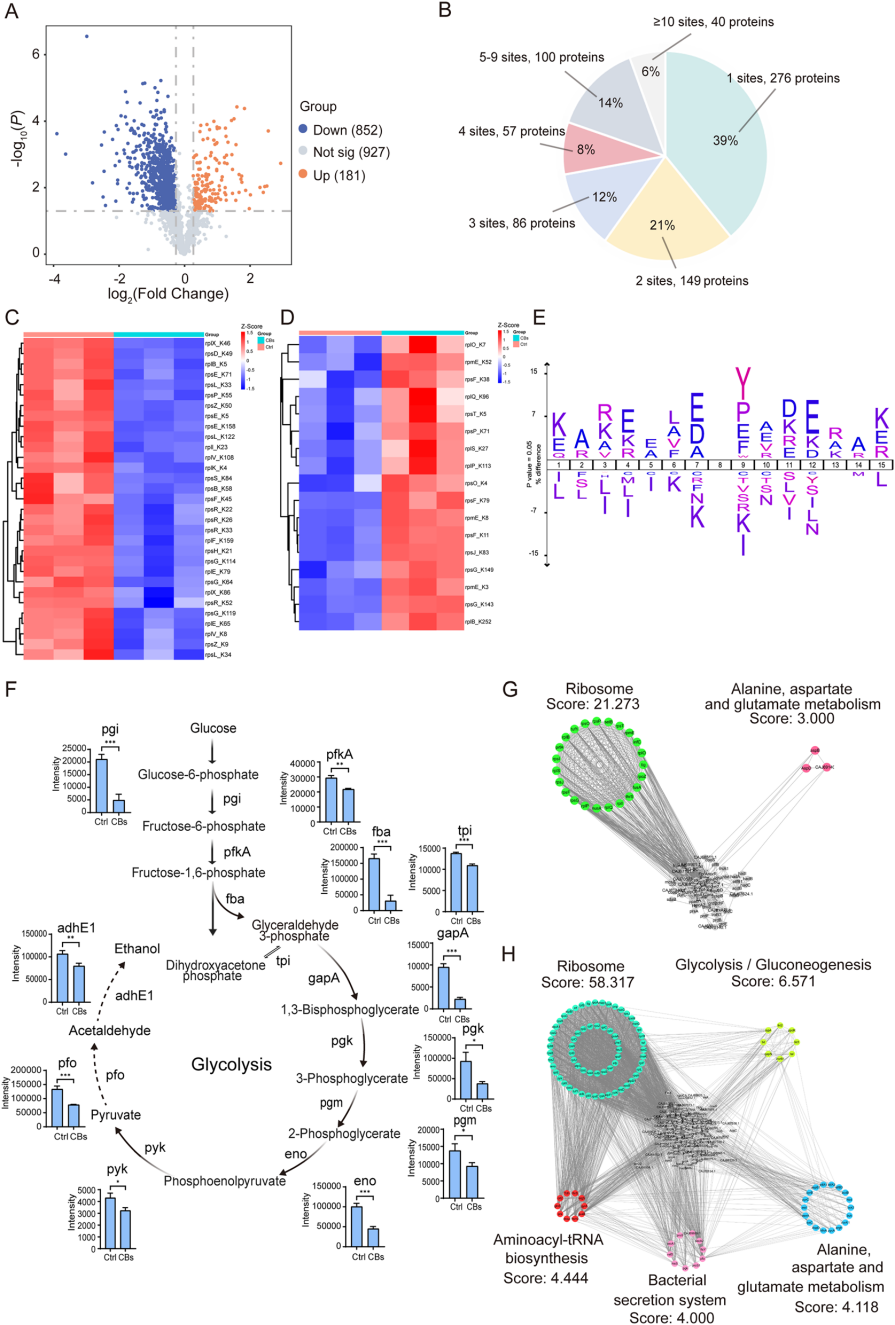
Global acetylome profiling of *C. difficile* in response to CBs. **A** Volcano plot of acetylated peptides (*p* < 0.05, FC > 1.2). **B** Distribution of 2,266 acetylation sites among 708 proteins. (C, D) Heatmaps of lysine-acetylated proteins upregulated **C** or downregulated **D** in ribosomal pathways. **E** Amino acid propensity analysis of the sequences flanking acetylation sites. **F** Glycolysis pathway annotated with key enzymes: pfkA, ATP-dependent 6-phosphofructokinase; fba, fructose-1,6-bisphosphate aldolase; tpi, triosephosphate isomerase; gapA, glyceraldehyde-3-phosphate dehydrogenase; pgk, phosphoglycerate kinase; pgm, phosphoglucomutase; eno, enolase; pyk, pyruvate kinase; pfo, pyruvate ferredoxin oxidoreductase; adhE1, aldehyde-alcohol dehydrogenase. **G**, **H** PPI networks of hyperacetylated (**G**) and hypoacetylated (**H**) proteins. Data represent mean ± SD (ns, *p* > 0.05; *, *p* < 0.05; **, *p* < 0.01; ***, *p* < 0.001; ****, *p* < 0.0001; Student’s t-test)

Utilizing high-specificity immunoprecipitation antibodies, we conducted comprehensive systems-level acetylome profiling following CBs exposure, which included detailed site identification and pathway annotation. Our quantitative proteomic analysis identified 852 downregulated and 181 upregulated proteins (Fig. 5A, Table S5). We detected 2266 acetylated peptides corresponding to 708 unique proteins (Fig. 5B). Importantly, these lysine-acetylated DEPs exhibited a strong association with ribosomal components. In response to cellular stress, characterized by the heterogeneity of ribosomes, cells strategically downregulate the synthesis of 31 core ribosomal proteins to conserve energy or adapt to stress (Fig. 5C), while concurrently upregulating 17 ribosomal protein variants to assemble functionally specialized ribosomes tailored to specific stress conditions (Fig. 5D). The analysis of sequence motifs at acetylation sites demonstrated notable positional preferences, characterized by an enrichment of glutamate at the -1 position and a predominance of lysine at the -7-position relative to the modified residues (Fig. 5E). Metabolic profiling indicated a decreased abundance of glycolysis enzymes in samples treated with CBs, with nearly complete coverage of the glycolysis pathway components (Fig. 5F). Additionally, PPI network analysis corroborated the presence of ribosomal-related networks among the DEPs (Fig. 5G, H). Thus far, our findings elucidate the intricate relationship between protein acetylation and *C. difficile*.

### Functional characterization of lysine residues in glycolysis enzymes FBA

**Fig. 6:**
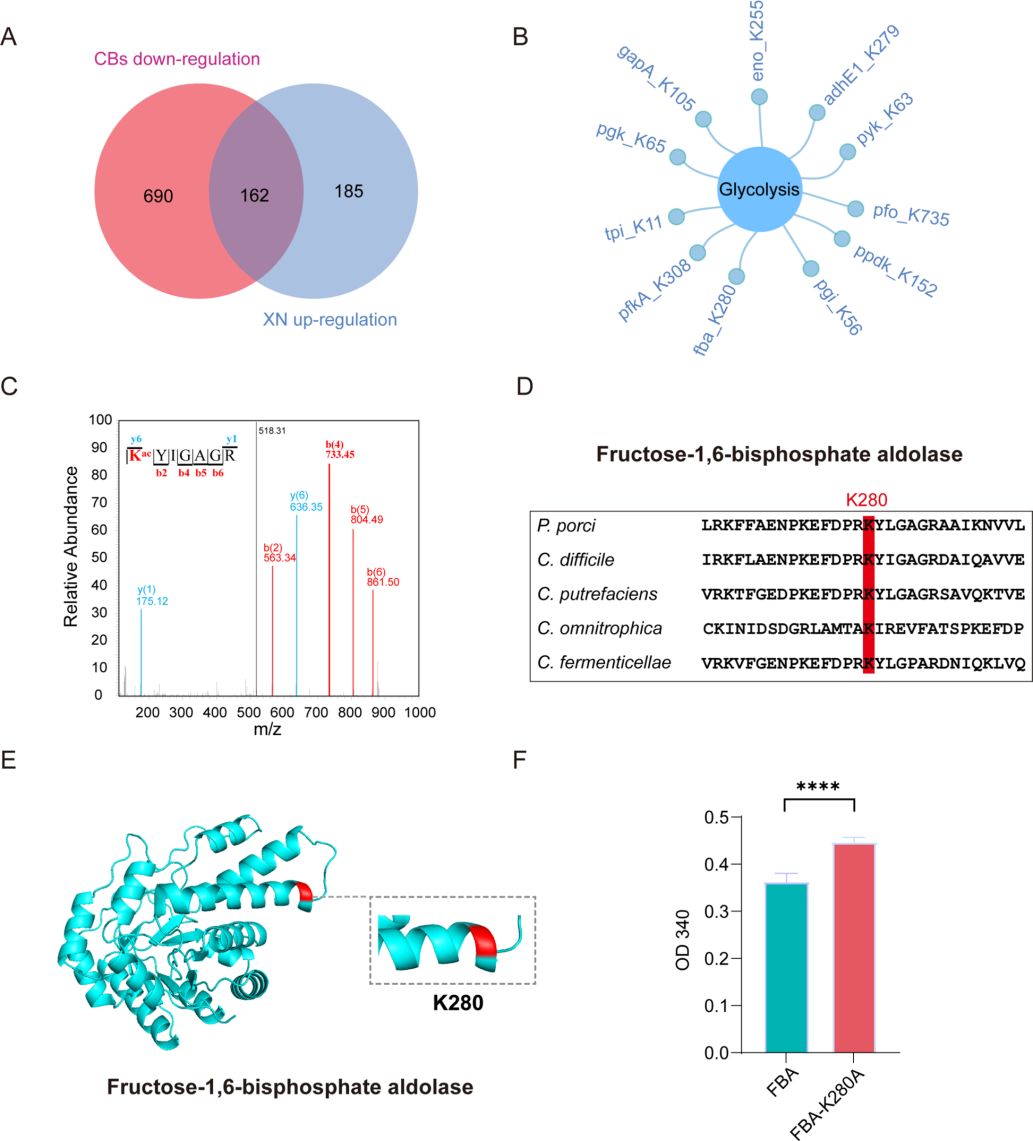
Functional characterization of lysine residues in glycolysis enzymes FBA. **A** Venn diagram of acetylated proteins downregulated by CBs and upregulated by XN. **B** Acetylated proteins associated with the glycolysis pathway. **C** MS/MS spectra of FBA peptides containing acetylated lysine (K280), with “b” and “y” ions indicating N-terminal and C-terminal fragments generated by peptide bond cleavage. **D** Cross-species sequence alignment with conserved lysine residue highlighted in red. **E** Three-dimensional localization of lysine residues (K280) in crystal structure visualized using PyMOL. **F** Relative enzymatic activities of wild-type and mutant substrates. Data represent mean ± SD (ns, p > 0.05; *, p < 0.05; **, p < 0.01; ***, p < 0.001; ****, p < 0.0001; Student’s t-test)

Significantly, our observations indicate that both XN and CBs interventions consistently target glycolysis pathway proteins in *C. difficile* via Kac. Elucidating the functional implications of these proteins remains a considerable challenge. Our proteomic analysis identified 162 acetylation sites with altered levels under both treatments (Fig. 6A, Fig. S5). Notably, 11 of these shared modification sites were located on key glycolysis enzymes, which are known to catalyze essential biochemical reactions (Fig. 6B). This finding suggests that acetylation modifications may coordinately regulate *C. difficile* energy metabolism through a multi-enzyme regulatory mechanism.

Given the central role of FBA in the glycolytic pathway and its well-established significance as a target in metabolic engineering [42], we selected it as a representative subject for subsequent mechanistic investigation. Using high-resolution mass spectrometry, we identified specific acetylation at lysine 280 (K280) of FBA in *C. difficile* (Fig. 6C). Bioinformatics analysis has demonstrated the evolutionary conservation of the acetylation site across a range of bacterial species (Fig. 6D). To elucidate the structural implications of the modification, we conducted a three-dimensional localization analysis of the acetylated lysine residue (K280) within the crystal structure of FBA using PyMOL software (Fig. 6E). The findings from this structural biology investigation indicate that the conserved acetylation site may be integral to the preservation of enzymatic structural integrity and catalytic functionality. To evaluate the functional significance of acetylation at lysine residue K280, we engineered single-point mutants by substituting these lysines with alanine (K280A) and subsequently assessed the enzymatic activities of FBA. Our findings revealed a diminished catalytic capacity in both the K280A mutants (Fig. 6F), thereby confirming the critical role of the residue in maintaining proper enzymatic function in *C. difficile*.

## Discussion

CDI, a potentially life-threatening gastrointestinal disorder, poses particularly severe clinical challenges when it coexists with IBD, significantly compromising patient outcomes. This study explored the interactions between natural products and microbes, as well as microbe-microbe interactions, through PTMs to demonstrate the therapeutic potential of XN and CBs against CDI. Our results demonstrate the therapeutic potential of XN against CDI in an IBD-relevant context and simultaneously provide a rationale for the further investigation of XN and CBs individually as potential therapeutic agents against CDI. Specifically, our results indicate that XN significantly mitigates *C. difficile*-induced cellular damage in chemically-induced Caco-2 colitis models. The compound effectively suppresses the aberrant activation of key inflammatory signaling pathways while maintaining the integrity of the intestinal epithelial barrier, collectively contributing to its potent enteroprotective effects. In contrast to conventional probiotic studies that employ whole bacterial administration [22], our experimental design incorporated an innovative approach using probiotic culture supernatants. This method allows for the direct assessment of bioactive metabolites while minimizing confounding variables associated with the application of live bacteria. While the established bioactivity of butyrate provides the rationale for using *C. butyricum*, our work specifically elucidates the proteomic response of *C. difficile* to CBs as a composite metabolite preparation. In this study, exposure to XN or CBs significantly reduced the pathogenic potential of *C. difficile* in infection models. Both interventions exhibited strong antibacterial effects through multiple mechanisms, including the reduction of viable bacterial counts, interference with colonization processes, and suppression of key virulence factor production. These findings align with existing evidence that hop-derived flavonoids disrupt host–pathogen interactions and virulence transcription in Gram-positive pathogens [43, 44]. Proteomic profiling revealed that both XN and CBs substantially alter the proteomic landscape of *C. difficile*. Mechanistically, XN impairs bacterial virulence by simultaneously inhibiting flagellar biosynthesis and disrupting oxidative phosphorylation pathways. Conversely, CBs primarily exerts its antimicrobial activity by suppressing glycolysis flux, leading to metabolic dysfunction. These results provide molecular-level insights into the diverse anti-infective mechanisms of both compounds against *C. difficile*.

Kac is an evolutionarily conserved regulatory PTM observed across all domains of life, from bacteria to humans. Recent research has underscored the pivotal role of acetylation in the context of bacterial infections. Utilizing *Salmonella* as a model pathogen, studies have shown that the dynamic acetylation of the PhoP protein within macrophages significantly influences *Salmonella* virulence [45]. In the current study, we conducted a comprehensive characterization of the Kac landscape in *C. difficile*, identifying that intervention with XN and CBs induces extensive Kac modifications. This has led to the discovery of previously unannotated acetylation sites and proteins in this pathogen. Our findings indicate that XN and CBs primarily target glycolysis pathways in *C. difficile*, suggesting that metabolic modulation could serve as a novel therapeutic strategy against CDIs. Beyond acetylation, other SCFA-mediated acylations and phosphorylation also play key roles in bacterial physiology, adding complexity to PTM networks [46, 47]. Building on this work, future studies will aim to elucidate the specific acylation profiles induced by various SCFAs and systematically explore the interplay between acylation and phosphorylation. The comprehensive acetylation map of *C. difficile* developed in this study serves as a foundational framework for these investigations.

In this study, comparative proteomic analyses of *C. difficile* treated with XN or CBs revealed opposing modulation of the glycolysis pathway enzyme abundance. Quantitative analysis indicated significantly increased levels of key glycolysis enzymes in cultures exposed to XN, in contrast to the decreased abundance observed in cultures treated with CBs. As a central determinant of virulence, toxin production is intricately regulated by nutritional signals, particularly the availability of carbon sources [48]. Most carbon sources that suppress toxin gene expression are internalized by bacterial cells through the phosphotransferase system (PTS) [49]. This observation implies that the regulatory effects on toxin transcription are associated with carbon catabolite repression (CCR) [50]. CCR facilitates the preferential consumption of easily metabolized carbon sources, such as glucose, by bacteria when multiple carbohydrates are available, thereby enhancing metabolic efficiency. In Gram-positive bacteria, CCR is predominantly regulated by the pleiotropic regulator CcpA, which generally acts as a positive regulator of glycolysis pathways [48]. The presence of glucose or other rapidly metabolized carbon sources in complex growth media leads to the suppression of toxin production, a phenomenon known as the glucose effect, which has been documented across various *C. difficile* strains [50]. This conservation among strains indicates the presence of a universal mechanism that links glucose availability to toxin regulation. Notably, glucose-mediated inhibition occurs at the transcriptional level, aligning with its suppression of *tcdR* expression [51]. These findings collectively indicate that both XN and CBs reduce toxin production through opposing modulation of central carbon metabolism. In *C. difficile* treated with XN, toxin production is suppressed at the protein level via upregulation of glycolysis and enhanced glucose utilization. In contrast, CBs inhibit glycolysis and initiate a broader shift toward catabolic remodeling. Previous RNA-seq analysis of the *C. difficile* used in this study revealed that butyrate (a major component of CBs) significantly downregulates genes associated with secondary metabolite biosynthesis, amino acid metabolism, and carbon metabolism. Butyrate exposure prompts metabolic reprogramming, characterized by the preferential upregulation of PTS transporters for alternative carbohydrates, such as mannose, lactose, and fructose, while concurrently suppressing glucose-centric metabolic pathways [52]. This metabolic shift provides a mechanistic explanation for the observed suppression of glycolysis following CBs treatment.

Polyphenolic compounds derived from natural sources have been extensively studied for their diverse mechanisms in inhibiting the pathogenesis of *C. difficile*. Recent investigations have revealed that the aglycone components of polyphenols possess novel functions in selectively targeting and suppressing this human intestinal pathogen [53]. Simultaneously, probiotics have emerged as a promising adjunctive therapeutic approach for managing gut microbiota dysbiosis induced by *C. difficile*. Clinical studies involving patients with IBD have highlighted the therapeutic potential of butyrate-producing bacterial species, such as *Faecalibacterium prausnitzii*, demonstrating their dual capacity for butyrate biosynthesis and immunomodulation [54, 55]. Based on these findings, our forthcoming research will utilize a modification-structure–function analytical framework to systematically delineate the multidimensional interactions among natural products, PTMs, and metabolic pathways across a variety of compounds. This approach aims to aid in the identification of novel therapeutic targets that could potentially act synergistically with XN. Furthermore, by employing integrated proteomic and PTM profiling, we will perform comparative analyses of the regulatory mechanisms utilized by butyrate-producing bacteria in contrast to CB in host-microbe interactions, with a particular focus on their differential modulation of the PTM profile of *C. difficile*.

While our findings indicate the promising anti-*C. difficile* efficacy of XN and CBs, we acknowledge certain limitations of this study. While the DSS-induced Caco-2 model provided a controlled platform for delineating the mechanisms of XN and CBs, it represents a simplified system relative to the intricate intestinal microenvironment in vivo. Further elucidation of their therapeutic effects through physiologically relevant colitis animal models is necessary and, importantly, the direct molecular targets of these compounds should be delineated via targetomic approaches. Such studies would offer compelling experimental evidence supporting the clinical potential of XN and CB formulations as novel therapeutic interventions for CDI.

Collectively, our results demonstrate that XN and CBs exhibit significant efficacy against CDI. The comprehensive dataset generated provides valuable protein-level insights for genetic and molecular biological investigations. These findings strongly advocate for the development of protein-targeted antimicrobial strategies as a promising therapeutic approach for combating CDI.

## Conclusions

Kac dynamics disrupt glycolytic flux in *C. difficile*, leading to impaired metabolic enzyme activity. This modulation effectively attenuates bacterial virulence by targeting central metabolic pathways. Strategic manipulation of PTMs offers a novel approach to control bacterial pathogenicity.

## Supplementary Information

Supplementary Material 1.** Fig. S1** Validation of key findings by protein abundance analysis. **A** Detection of protein abundance related to the inflammatory NFκB pathway. **B**, **C** Validation of protein abundance for key steroid biosynthesis enzymes Q16850 and P37268 in the DSS+CD group: Q16850, Lanosterol 14-alpha demethylase; P37268, Squalene synthase. **D** Effect of XN intervention on the protein abundance of *C. difficile *toxin *tcdA*. Data represent mean ± SD (ns, *p* > 0.05; *,* p* < 0.05; **,* p* < 0.01; ***,* p* < 0.001; ****, *p* < 0.0001). **Fig. S2** Pathway enrichment analysis of 662 DEPs in the XN group. **A** Annotated keywords. **B** Biological process analysis. **Fig. S3 **The ratio of CBs to *C. difficile *was determined by OD₆₀₀ measurement. Data represent mean ± SD (ns, *p* > 0.05; *, *p* < 0.05; **, *p* < 0.01; ***, *p* < 0.001; ****, *p* < 0.0001). **Fig. S4** Abundance profiles of acetylation-related enzymes in *C. difficile *treated with XN or CBs. **A** Deacetylases abundance: Q183F9, peptidoglycan-N-acetylglucosamine deacetylase; Q18BG4, peptidoglycan-N-acetylglucosamine deacetylase; Q180V0, N-acetylglucosamine-6-phosphate deacetylase. **B** Acetyltransferases abundance: Q189M1, acetyltransferase; Q18B70, acetyltransferase CD1211; Q184G5, acetyltransferase; Q186Y1, N-acetyltransferase GCN5. Data represent mean ± SD (ns, *p* > 0.05; *, *p* < 0.05; **, *p* < 0.01; ***, *p* < 0.001; ****, *p* < 0.0001; Student’s t-test). **Fig. S5** Functional assessment of glycolysis in *C. difficile *following treatment with XN or CBs. **A** Glucose levels after treatment with XN (left) or CBs (right). **B** Lactate levels after treatment with XN (left) or CBs (right). Data represent mean ± SD (ns, *p* > 0.05; *, *p* < 0.05; **, *p* < 0.01; ***, *p* < 0.001; ****, *p* < 0.0001; Student’s t-test)

Supplementary Material 2. Table S1 List of regulated proteins in Caco-2 in response to xanthohumol

Supplementary Material 3. Table S2 List of regulated proteins in *C. difficile *in response to xanthohumol

Supplementary Material 4. Table S3 List of regulated proteins in *C. difficile *in response to CBs

Supplementary Material 5. Table S4 Xanthohumol-regulated lysine acetylation sites in *C. difficile*

Supplementary Material 6. Table S5 CBs-regulated lysine acetylation sites in *C. difficile*

Supplementary Material 7. Table S6 Primers used in this study

## Data Availability

The datasets generated and analysed during the study are available in the iProX Consortium with ID：IPX0012980001.

## Abbreviations

*C. difficile*: *Clostridioides difficile*
CDI: *Clostridioides difficile* Infection
IBD: Inflammatory bowel disease
XN: Xanthohumol
SCFAs: Short-chain fatty acids
*C. butyricum*: *Clostridium butyricum*
CBs: *Clostridium butyricum* Supernatant
PTMs: Post-translational modifications
LC–MS/MS: Liquid chromatography-tandem mass spectrometry
DSS: Dextran sulfate sodium
PCA: Principal component analysis
KEGG: Kyoto encyclopedia of genes and genomes
CFSE: Carboxyfluorescein succinimidyl ester
TMT: Tandem mass tag
FBA: Fructose-1,6-bisphosphate aldolase
Kac: Lysine Aacetylation
FDR: False discovery rate
MCODE: Molecular complex detection
FC: Fold change
DEPs: Differentially expressed proteins
PPI: Protein–protein interaction
IPTG: Isopropyl-β-D-thiogalactopyranoside
PBS: Phosphate-buffered saline
TFA: Trifluoroacetic acid
LB: Lysogeny Broth
PTS: Phosphotransferase system
CCR: Carbon catabolite repression
