# Supplementary material for: Proteomic antibacterial characterization of flavonoid xanthohumol and probiotic Clostridium butyricum on pathogenic Clostridioides difficile
Source: Chin Med. 2026 Feb 3;21:64. doi: 10.1186/s13020-026-01343-x (PMC12870184; doi:10.1186/s13020-026-01343-x)
Supplement: Supplementary file 1 — Supplementary Material 1. Fig. S1 Validation of key findings by protein abundance analysis. A Detection of protein abundance related to the inflammatory NFκB pathway. B, C Validation of protein abundance for key steroid biosynthesis enzymes Q16850 and P37268 in the DSS+CD group: Q16850, Lanosterol 14-alpha demethylase; P37268, Squalene synthase. D Effect of XN intervention on the protein abundance of C. difficile toxin tcdA. Data represent mean ± SD (ns, p > 0.05; *, p < 0.05; **, p < 0.01; ***, p < 0.001; ****, p < 0.0001). Fig. S2 Pathway enrichment analysis of 662 DEPs in the XN group. A Annotated keywords. B Biological process analysis. Fig. S3 The ratio of CBs to C. difficile was determined by OD₆₀₀ measurement. Data represent mean ± SD (ns, p > 0.05; *, p < 0.05; **, p < 0.01; ***, p < 0.001; ****, p < 0.0001). Fig. S4 Abundance profiles of acetylation-related enzymes in C. difficile treated with XN or CBs. A Deacetylases abundance: Q183F9, peptidoglycan-N-acetylglucosamine deacetylase; Q18BG4, peptidoglycan-N-acetylglucosamine deacetylase; Q180V0, N-acetylglucosamine-6-phosphate deacetylase. B Acetyltransferases abundance: Q189M1, acetyltransferase; Q18B70, acetyltransferase CD1211; Q184G5, acetyltransferase; Q186Y1, N-acetyltransferase GCN5. Data represent mean ± SD (ns, p > 0.05; *, p < 0.05; **, p < 0.01; ***, p < 0.001; ****, p < 0.0001; Student’s t-test). Fig. S5 Functional assessment of glycolysis in C. difficile following treatment with XN or CBs. A Glucose levels after treatment with XN (left) or CBs (right). B Lactate levels after treatment with XN (left) or CBs (right). Data represent mean ± SD (ns, p > 0.05; *, p < 0.05; **, p < 0.01; ***, p < 0.001; ****, p < 0.0001; Student’s t-test) [file 13020_2026_1343_MOESM1_ESM.docx]

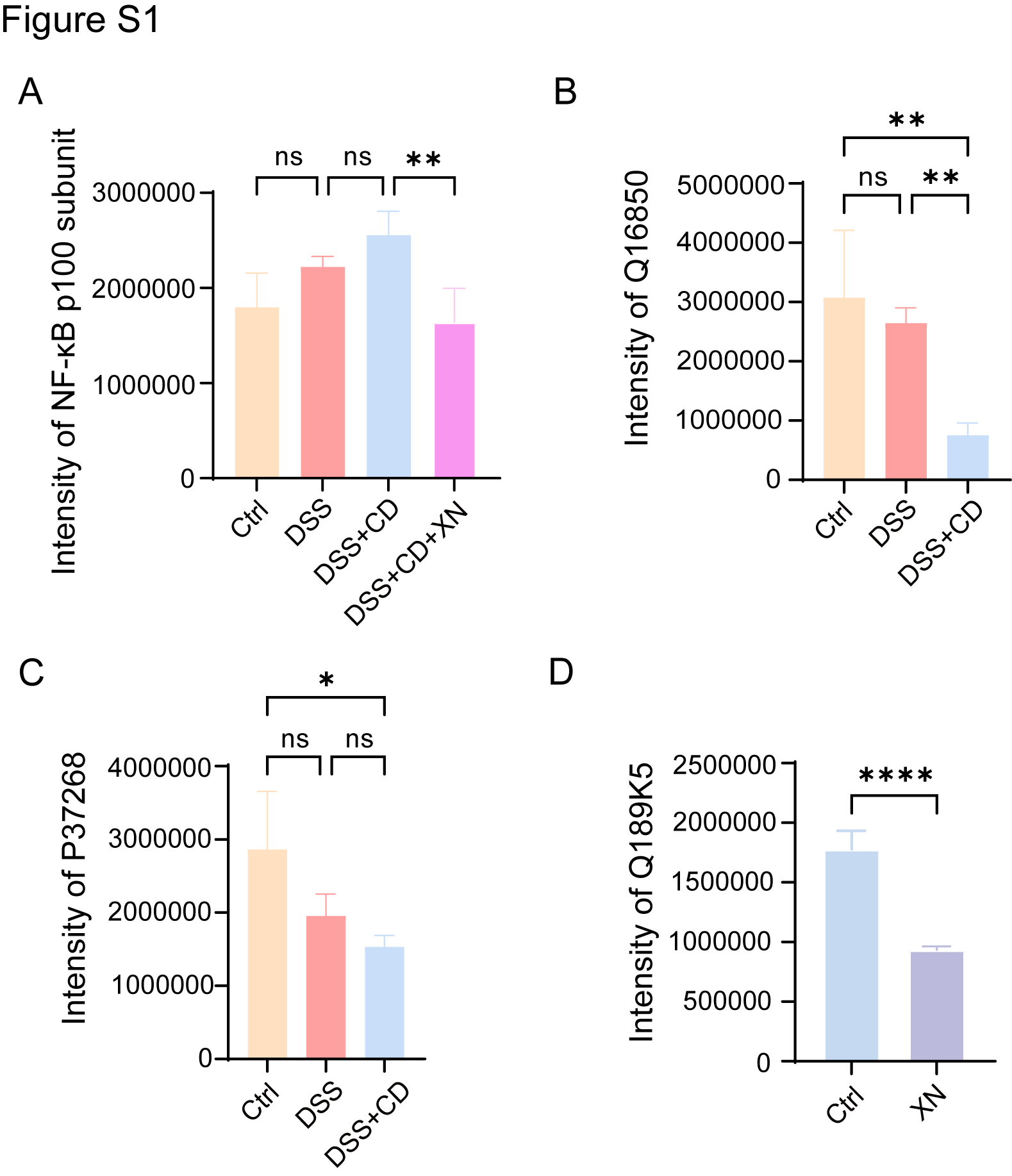


**Fig. S1** Validation of key findings by protein abundance analysis. (A) Detection of protein abundance related to the inflammatory NF‑κB pathway. (B, C) Validation of protein abundance for key steroid biosynthesis enzymes Q16850 and P37268 in the DSS+CD group: Q16850, Lanosterol 14-alpha demethylase; P37268, Squalene synthase. (D) Effect of XN intervention on the protein abundance of *C. difficile* toxin *tcdA*. Data represent mean ± SD (ns, *p* > 0.05; *, *p* < 0.05; **, *p* < 0.01; ***, *p* < 0.001; ****, *p* < 0.0001).


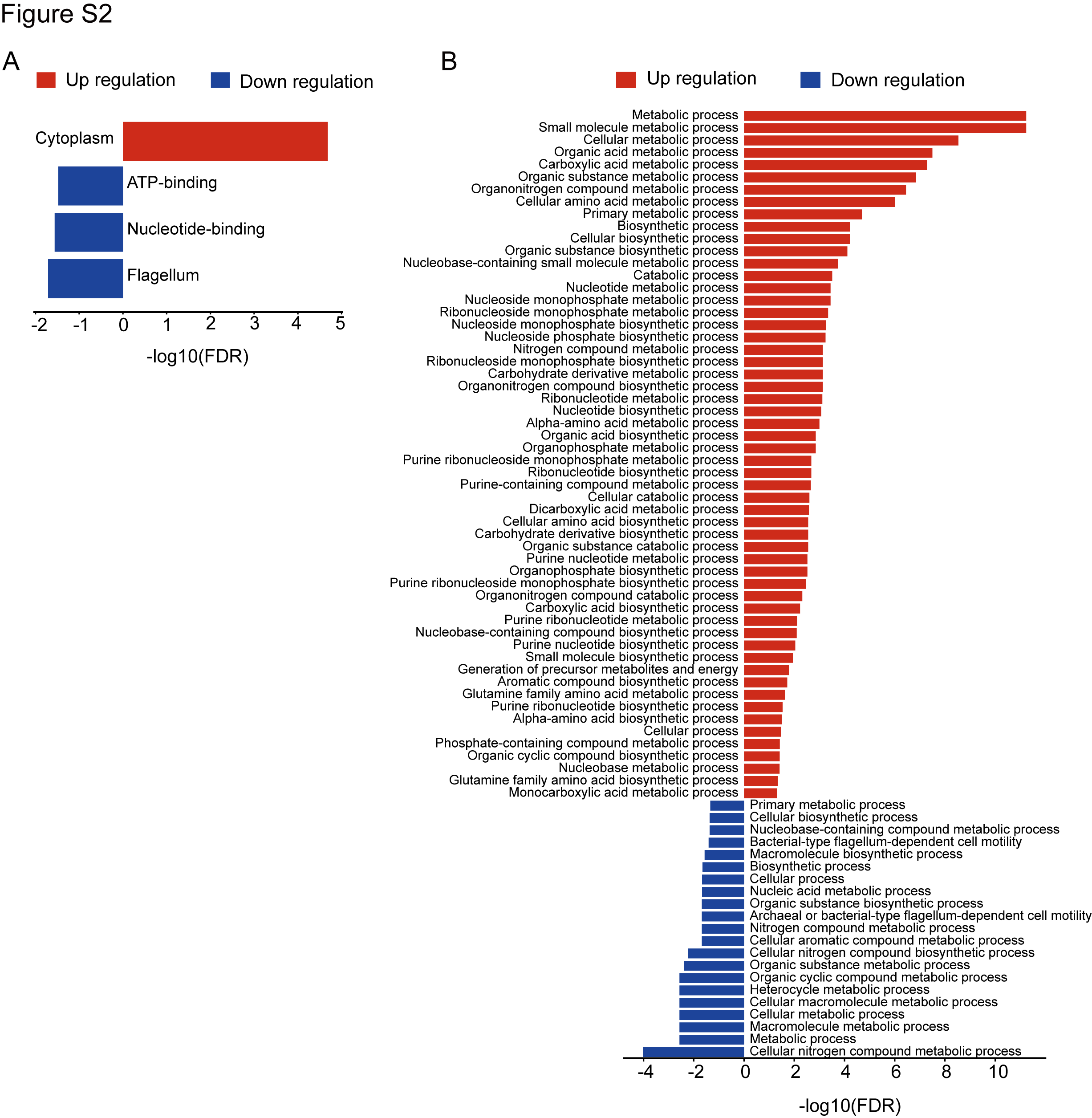


**Fig. S2** Pathway enrichment analysis of 662 DEPs in the XN group. (A) Annotated keywords. (B) Biological process analysis.


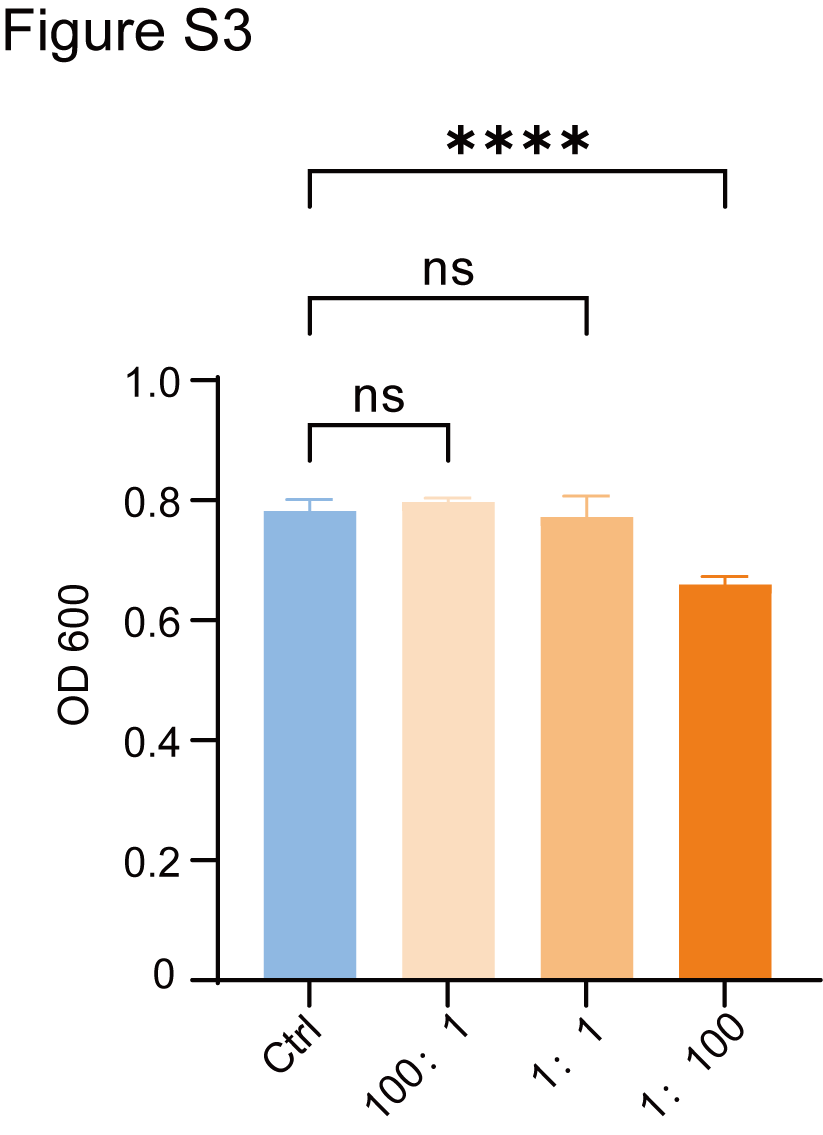


**Fig. S3** The ratio of CBs to *C. difficile* was determined by OD₆₀₀ measurement. Data represent mean ± SD (ns, *p* > 0.05; *, *p* < 0.05; **, *p* < 0.01; ***, *p* < 0.001; ****, *p* < 0.0001).


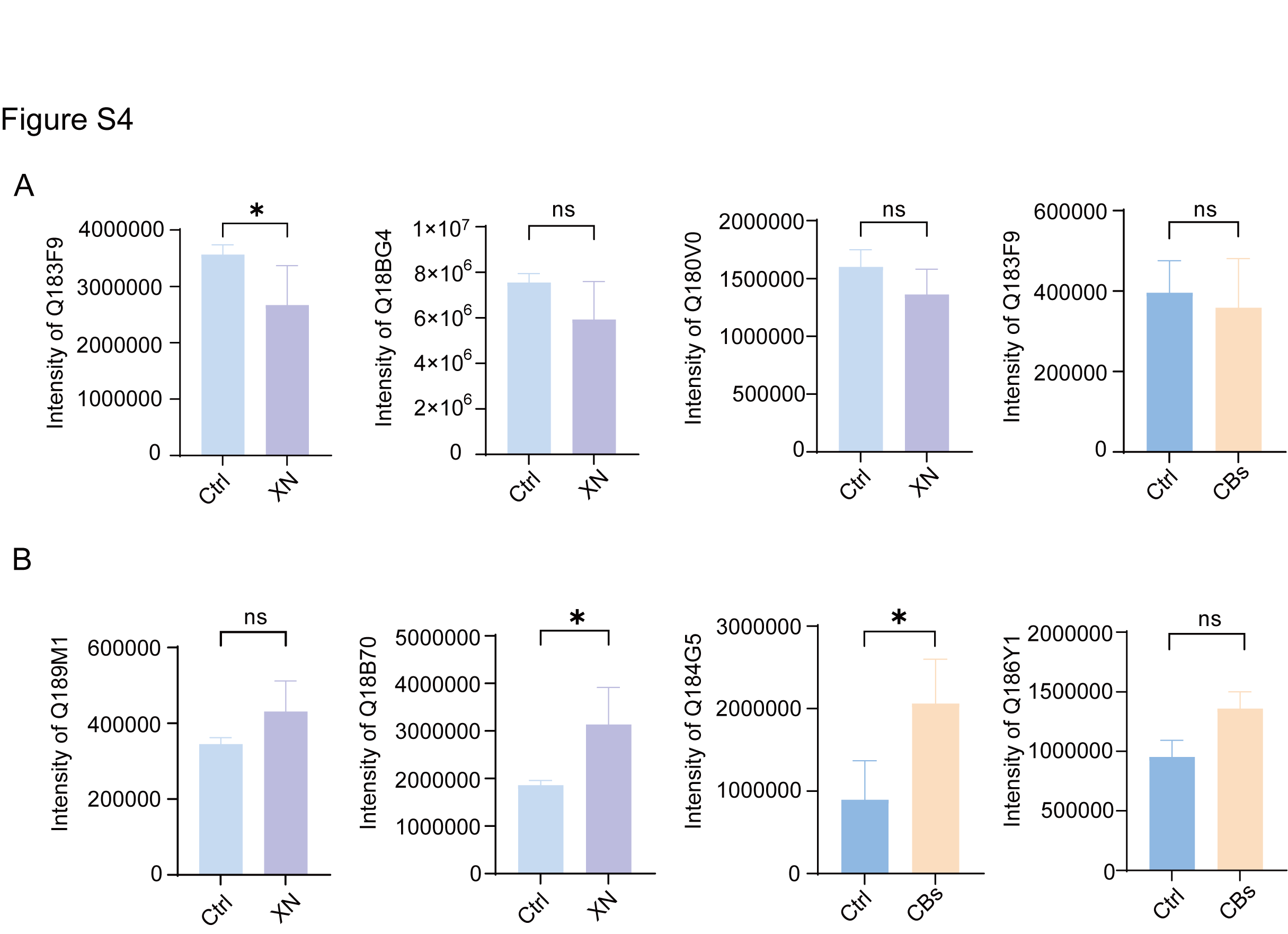


**Fig. S4** Abundance profiles of acetylation-related enzymes in *C. difficile* treated with XN or CBs. (A) Deacetylases abundance: Q183F9, peptidoglycan-N-acetylglucosamine deacetylase; Q18BG4, peptidoglycan-N-acetylglucosamine deacetylase; Q180V0, N-acetylglucosamine-6-phosphate deacetylase. (B) Acetyltransferases abundance: Q189M1, acetyltransferase; Q18B70, acetyltransferase CD1211; Q184G5, acetyltransferase; Q186Y1, N-acetyltransferase GCN5. Data represent mean ± SD (ns, *p* > 0.05; *, *p* < 0.05; **, *p* < 0.01; ***, *p* < 0.001; ****, *p* < 0.0001; Student’s t-test).


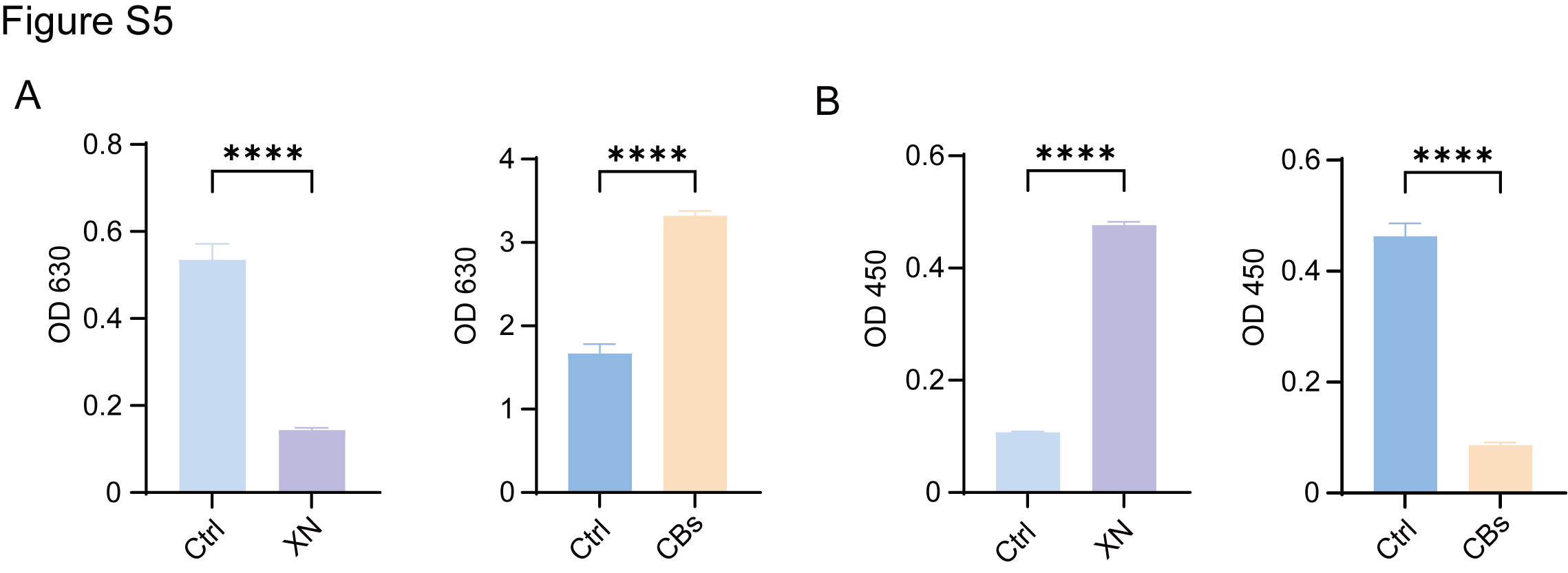


**Fig. S5** Functional assessment of glycolysis in *C. difficile* following treatment with XN or CBs. (A) Glucose levels after treatment with XN (left) or CBs (right). (B) Lactate levels after treatment with XN (left) or CBs (right). Data represent mean ± SD (ns, *p* > 0.05; *, *p* < 0.05; **, *p* < 0.01; ***, *p* < 0.001; ****, *p* < 0.0001; Student’s t-test).
